# Bioactive carbon improves nitrogen fertiliser efficiency and ecological sustainability

**DOI:** 10.1038/s41598-020-60024-3

**Published:** 2020-02-24

**Authors:** Peter Espie, Haley Ridgway

**Affiliations:** 1 AgScience Research, 333 Chain Hills Road, R.D. 1., Dunedin, 9076 New Zealand; 2 Lincoln University, Ellesmere Junction Road, Lincoln, 7647 New Zealand

**Keywords:** Agroecology, Agroecology, Environmental chemistry

## Abstract

Agriculture’s most pressing challenge is raising global food production while minimising environmental degradation. Nutrient deficiencies, principally nitrogen (N), limit production requiring future increases in fertiliser use and risk to proximal non-agricultural ecosystems. We investigated combining humate with urea, globally the most widely used N-suppling fertiliser, in a four-year field study. Humate increased pasture yield by 9.8% more than urea and significantly altered soil microbial diversity and function. Humate increased N retention suggesting microbial sequestration may lower N leaching and volatilisation losses. Humic microbial bio-stimulation could feasibly increase fertiliser efficiency and development of ecologically sustainable agriculture.

## Introduction

It is estimated one billion people currently suffer from chronic hunger and that global food production needs to double when the global population will stabilise at around 9.8 billion in 2050^1,2^. Increases in agricultural output have been largely driven by raising the use-efficiency of labour, land, capital and other inputs, including fertilisers^3,4^, but further escalation in food demand will place increasing pressure on finite land and water resources, raising serious concerns over land use intensification and global food security^5–7^. The challenge of meeting sustainable food production while achieving the UN Millennium Development Goals of ending hunger and ecosystem protection is substantial^8–10^ and has been described as “a perfect storm”^11^.

Raising global food production will unavoidably require increases in fertiliser inputs, principally nitrogen (N) and phosphorus (P)^12,13^. N is essential in all biological processes as a primary component of nucleotides and proteins and is a principle factor determining ecosystem productivity. N supply is frequently insufficient in terrestrial systems for full realisation of potential plant growth, hence its fundamental importance in agricultural and horticultural production. Throughout history N fertiliser application has been used to increase the productive efficiency of land and it is a major component of the “Green Revolution” responsible for meeting global food demand for about half of the world’s population^12,14,15^.

Inert nitrogen comprises 78% of the atmosphere, comprising a substantial ecological resource, but is only made biologically available by lightning or, more commonly, by N-fixing micro-organisms. Development of industrial synthesis of ammonia using atmospheric N occurred early in the 20^th^ century leading to production of abundant and cheap fertilisers, principally urea, and subsequent widespread adoption of their use. Intensive use of agricultural synthetic N has significant environmental costs^16,17^. Nitrogen is highly mobile in soil solution and readily moves from agro-ecosystems via unmanaged pathways, influencing non-agricultural ecosystems^14^. Atmospheric N from agricultural and other land uses affects the global climate, comprising 81% of human derived nitrous oxide (N_2_O) emissions during 2007–2016 and contributing around 28% of the total net anthropogenic greenhouse gas emmissions^18^. Localised atmospheric N deposition also affects natural biodiversity and ecosystem function^14^. Leached inorganic N creates major aquatic environmental problems by a variety of pathways, acidification of unbuffered freshwater ecosystems and eutrophic stimulation of primary producers in freshwater and marine systems, which can result in anoxia or toxicity and significant biological impacts. The required increase in global agricultural fertiliser is forecast to cause 2.4- to 2.7-fold increases in nitrogen- and phosphorus-driven eutrophication of terrestrial, freshwater, and near-shore marine ecosystems, with significant loss of ecosystem services^19^. While there is no simple solution, scientific and technological innovation remain a key strategy for implementing sustainable intensification, defined as producing more food from the same land area while reducing environmental impacts^20^.

It has long been known that soil microbial communities have a key role in mediating ecosystem processes, notably soil structure and nutrient turnover^21^, but developments in quantitative genomic analysis have facilitated approaches for tackling previously difficult investigations of plant-microbiome dynamics^22,23^. Recent studies have shown how closely microbial diversity and function are coupled with plant productivity via root exudates, nutrient cycling and uptake, plant growth processes and climate change^24–26^.

Humic substances are naturally occurring organic compounds arising from the decomposition of plant, animal and microbial residues and many studies have reported beneficial effects on plant processes and productivity^27–30^. Other studies have reported neutral or negative responses, uncertainty regarding modes of action and inconsistency in effectiveness^31–34^. Reviews repeatedly note the scarcity of evidence from robust long-term field trials, resulting in uncertainty regarding recommendation for commercial application^35–37^.

Advances in analytical technology have also open new approaches to investigate humic substance chemistry providing tools for explicit molecular evaluation of their bioactivity^38–40^. This has resulted in fundamental reinterpretation of previous analytical misinterpretations of humic substances^39,41^ and holds significant potential for clarifying understanding of response pathways, a major requirement for determining future practical applications.

Another technological innovation, particulate coating of N fertilisers, has recently been advocated as a potential solution for improving fertiliser efficiency, with studies reporting that coated urea improved N release and reduced N loss^42–44^.

We hypothesised that coating urea with humic carbon may act as a microbial bio-stimulant, improving plant nutrient acquisition. To test this we designed a field experiment in developed pasture grassland applying a standard rate of urea fertiliser, urea coated with humate and urea and P-supplying guano coated with humate. We measured monthly or bi-monthly herbage production over four growing seasons from 2014–2018 and assessed microbial diversity and activity microscopically and by DNA amplification for fungi, bacteria and gammaproteobacteria, bacteria with genes for nitrogen oxidising capability.

## Methods

### Study site

The study was located at Agland Farm, Mataura, New Zealand (46° 48′33S; 168° 48′04E) at 141 m above sea level in fertilised pasture, principally consisting of ryegrass (*Lolium perenne* L.) and white clover (*Trifolium repens* L.). The climate is temperate with a mean annual temperature of10.6 °C, ranging from 14.8 °C in summer to 4.6 °C in winter, and a mean annual precipitation of 916 mm (2015–2018). The soil is a Waimumu silt loam, rolling phase classified as an imperfectly to poorly drained Mottled Fragic Pallic/ Perched Gley Soil^45^ with moderate over slow permeability. The upper topsoil (0–15 cm depth) had a pH of 6.1, a total carbon content of 4.5%, a total nitrogen content of 0.30%, Olsen extractable phosphate of 7 mg/L, and a base saturation of 63.0%. The site has been used for over 50 years for pasture grazing and cultivated for fodder crop production. It was last fertilised 18 months before trial commencement with 125 kg/ha superphosphate and 1 tonne/ha lime.

### Materials

We used commercially available urea from Ballance Agri-Nutrients and Southern Humate, mined from the Kapuka K1b lignite seam at Waituna, Southland. Humate was fine ground to <2 mm and mixed with granular urea and lime immediately prior to application. Total carbon content averaged 56.45 ± 0.05% (standard error of the mean), humic acid 36.7 ± 2.0%, fulvic acid 2.03 ± 0.08% and cation exchange capacity (CEC) was 125.5 ± 0.0 me/100 g.

### Experimental design and sampling

We initially investigated pasture responses to five rates of humate with urea and guano in the first growing season (2014–2015) using a randomised complete design block design with eight fertiliser applications in five blocks, totalling 40 plots with 5 replications per treatment. Treatments were nil fertiliser, five rates of humate and urea applied monthly, and one rate of humate and urea applied bimonthly with or without gauno. Humate rates were 0, 2.5%, 5%, 10% and 20% of the weight of urea, applied initially as 60 kg/ha of urea with 6 kg/ha of lime on the 28^th^ October 2014 and then applied monthly as 50 kg/ha of urea with 5 kg/ha lime from November 2014 to February 2015. The bi-monthly 10% humate rate was applied ± 100 kg/ha guano. Total seasonal urea applied varied from 160 to 260 kg/ha per treatment.

In the following growing seasons we examined responses using urea with the two highest yielding rates of humate with or without lime. The experimental design was a randomised complete block with five fertilisers in five blocks with a split-plot lime treatment ± 5 kg/ha, totalling 40 plots with five (nil) or eight or nine main treatment replicates with four or five sub-treatment lime replicates Treatments were nil fertiliser without lime, urea at 50 kg/ha ± lime, urea at 50 kg/ha with 5 kg/ha humate (10%) ± lime, urea at 50 kg/ha with 10 kg/ha humate (20%) ± lime, and urea at 50 kg/ha plus 5 kg/ha humate and 50 kg/ha gauno ± lime. Fertilisers were applied ~ bimonthly four times each season between November and April, totalling 200 kg/ha urea in the second and third seasons but were only applied once, in spring of the last season (October 2017, totalling 50 kg/ha urea), to examine the longevity of fertiliser effects. Plot size was 10 m^2^ separated by 1 or 2 m buffer zones.

### Pasture production assessment

Pasture yield was determined by harvesting with rotary mower to a 4–6 cm cut height, except for 2–3 cm in April 2017, and weighed to an accuracy of 5 g. Herbage was evenly returned and fresh weight sub-samples were taken from every plot, weighed to an accuracy of 0.02 g and dried to constant weight for calculating dry matter (DM). There were five approximately monthly assessments during the initial growing season (November 2014 - March 2015), eight in the second (September 2015 – April 2016), six in the third (September 2016 – April 2017) and three ~bimonthly assessments in the final season, when determining the longevity of fertiliser effects (October 2017 – March 2018).

### Microscopic assessment of soil microbiology

Fertiliser effect on seasonal changes in fungal and bacterial abundance and activity were determined in spring immediately before fertiliser application (27^th^ October 2014) from a bulk sample of forty 2.5 ×x 7.5 cm topsoil cores, in summer (3^rd^ February 2015) from a bulk sample of four cores per plot and in autumn (20^th^ March 2015) from ten cores per plot, totalling 40 samples per assessment. Microbial biomass and activity was determined using epifluorescent and phase contrast-differential interference contrast microscopy by Soil Food Web New Zealand^46^.

### DNA assessment of soil microbiology

To assess fertiliser effect on microbiology after two seasons, three topsoil cores were extracted as previously from every plot on the 9^th^ January 2017, providing 40 bulked samples for DNA analysis, and frozen at −20 °C until laboratory processing. Soil was thoroughly mixed and a 12 gram sub sample was shaken with 50 ml of sterile water then placed into an ultrasonic bath for 3 minutes. A 2 ml extract was centrifuged for 15 minutes at 11,400 × *g*, the supernatant removed and the residue stored at −20 °C. DNA was extracted using DNeasy PowerSoil kit (Qiagen protocols), concentration measured by spectrophotometer using a Nanodrop™, and stored at −20 °C. This was replicated three times giving a total of 120 assays. Possible detection errors were checked, and if necessary corrected, by examination of replicate consistency.

Microbial DNA in soil samples was determined using Denaturing Gradient Gel Electrophoresis (DGGE) analysis by amplification using three PCR primer sets: the total bacterial communities identified using the V3 hypervariable region of the 16 S rRNA gene with primers 341 F GC and 518R^47^; the total fungal communities using the primer pair AU2 and AU4 and the PCR product from the primer pair AU2-AU4 used as template for a second nested PCR with primer pair FF390 and FR1-GC^47^,^48^; and the gammaproteobacteria communities using a nested PCR with primers (Gamma395F - Gamma871R and 518F GC - 785R)^49^. Diversity of ammonium oxidising bacteria was similarly determined using amplification of the ammonia monooxygenase subunit A gene (*amoA)* with primers amoA1F-GC and amoA2IR^50^. Phoretix 1D Pro software measured DNA band profiles.

### Statistical analysis

The statistics package R 3.6.2 was used for data analysis^51^. The Shapiro-Wilk and Kolmogorov- Smirnov tests examined assumptions of normality for modelled distributions. If required, the data were further analysed using negative binomial general linear models. Analysis of variance and regression using linear or generalised linear models was used to test fertiliser effects with Tukey tests for pairwise comparisons. Analyses were also run with Welch F or Kruskal-Wallis tests not assuming equal variances or normal distributions. Chi square and Fisher’s exact test of independence were used to test the probability of observed responses. Fertiliser effect on microbial population composition was assessed by multivariate canonical correspondence analysis and multivariate general linear models with resampling using the vegan and mvabund packages^52^,^53^.

## Results

**Figure 1:**
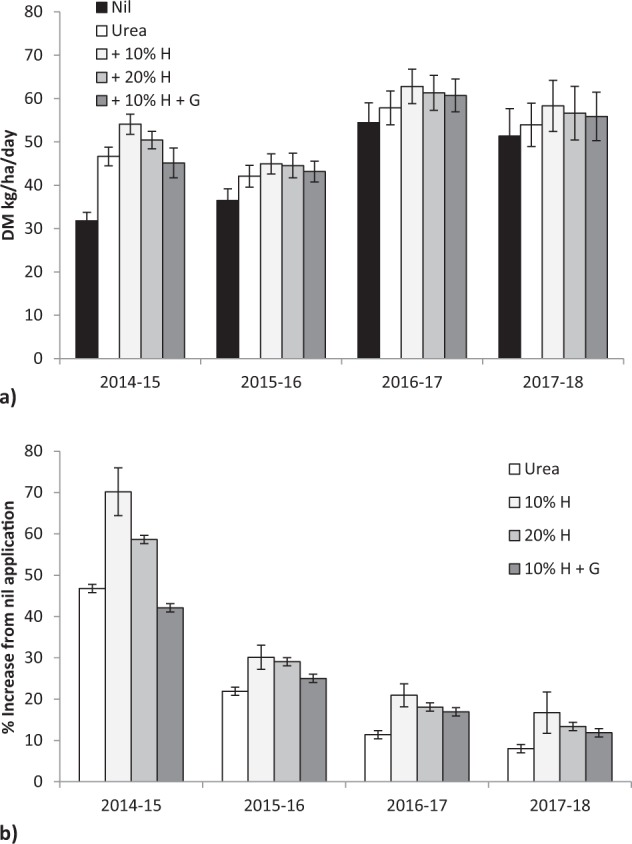
(**a**) Effect of urea, humate (H) and guano (G) addition to urea on pasture dry matter (DM) production 2014–15, 2015–16, 2016–17, 2017–18 growing seasons; (**b**) Change in production relative to unfertilised grassland, mean ± standard error.

Fertiliser application significantly increased pasture dry matter (DM) production, averaged across four growing seasons (F = 6.06, P < 0.00001), with a significant difference between seasons (F = 26.39, P < 0.00001) with no fertiliser x season interaction (F = 0.32, P < 0.98; Fig. 1a). In the first growing season (2014–15) urea increased average production by 47% compared with nil fertiliser application in unfertilised grassland, urea with 10% humate by 70%, urea with 20% humate by 59% and urea with 10% humate and gauno by 42% (F = 13.4, P < 0.001, Fig. 1b). By the final growing season (2017–18) urea increased production by 8%, urea with 10% humate by 17%, urea with 20% humate by 13% and urea with 10% humate and gauno by 12% (F = 1.08, P < 0.37, Fig. 1d). In the twenty two assessments between 2014 to 2018 addition of 10% humate to urea gave greater production than urea in every assessment (Chi square = 44.0, P < 0.0001; Fisher’s Exact Test P < 0.0001) and 20% humate gave greater production in 20 assessments (Chi square = 36.7, P < 0.0001; Fisher’s Exact Test P < 0.0001). Lime addition did not significantly change production in any season, or with any fertiliser (54.9 vs 55.3 kg DM/ha/day; F = 42.4, P < 0.922).

Increasing humate rate significantly linearly increased production by March and September in the first year of the trial (DM = 0.256 * Humate % + 38.5, F = 4.49, P < 0.045; DM = 0.126 * Humate % + 7.4, F = 4.50, P < 0.045) with variability within treatments masking differentiation between linear and curvilinear responses.

**Figure 2:**
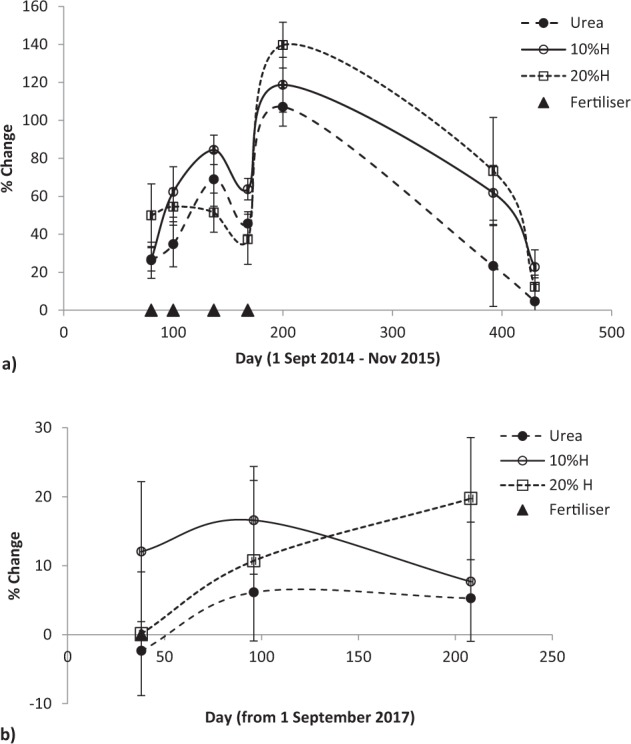
Effect of humate application rate on pasture production in March and September 2015 and 2014–2018, mean ± standard error. Longevity of urea and humate (H) effect on pasture production relative to unfertilised pasture (**a**) after summer application of the 2014–15 season to the start of the 2015–16 growing seasons and (**b**) after spring application in the 2017–18 season, mean ± standard error.

Fertiliser applied by summer of 2015 continued to affect pasture production after seven and a half months, though non-significantly (F = 2.45, P < 0.079), before production sharply fell towards unfertilised pasture a month later (Fig. 2a). In both the initial and final growing seasons (Fig. 2a,b) humate addition produced higher yields than from urea, notably in the spring of the 2015–16 growing season where the production difference widened from 6% and 16% (at the 10% and 20% humate rates) in the autumn of the 2014–15 season to 31% and 41% in early spring of the 2015–16 season. In the final growing season these humate rates lifted production above urea by 12% and 20% (Fig. 2b).

**Figure 3:**
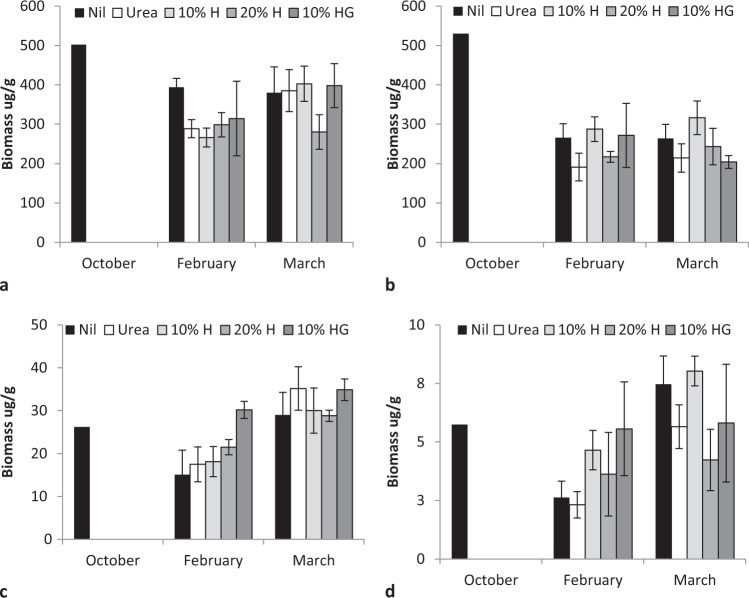
Effect of urea, humate (H) and guano (G) addition to urea on (**a**) total bacterial biomass and; (**b**) total fungal biomass (**c**) active bacterial biomass and (**d**) active fungal biomass, October 2014 to March 2015, mean ± standard error.

**Figure 4:**
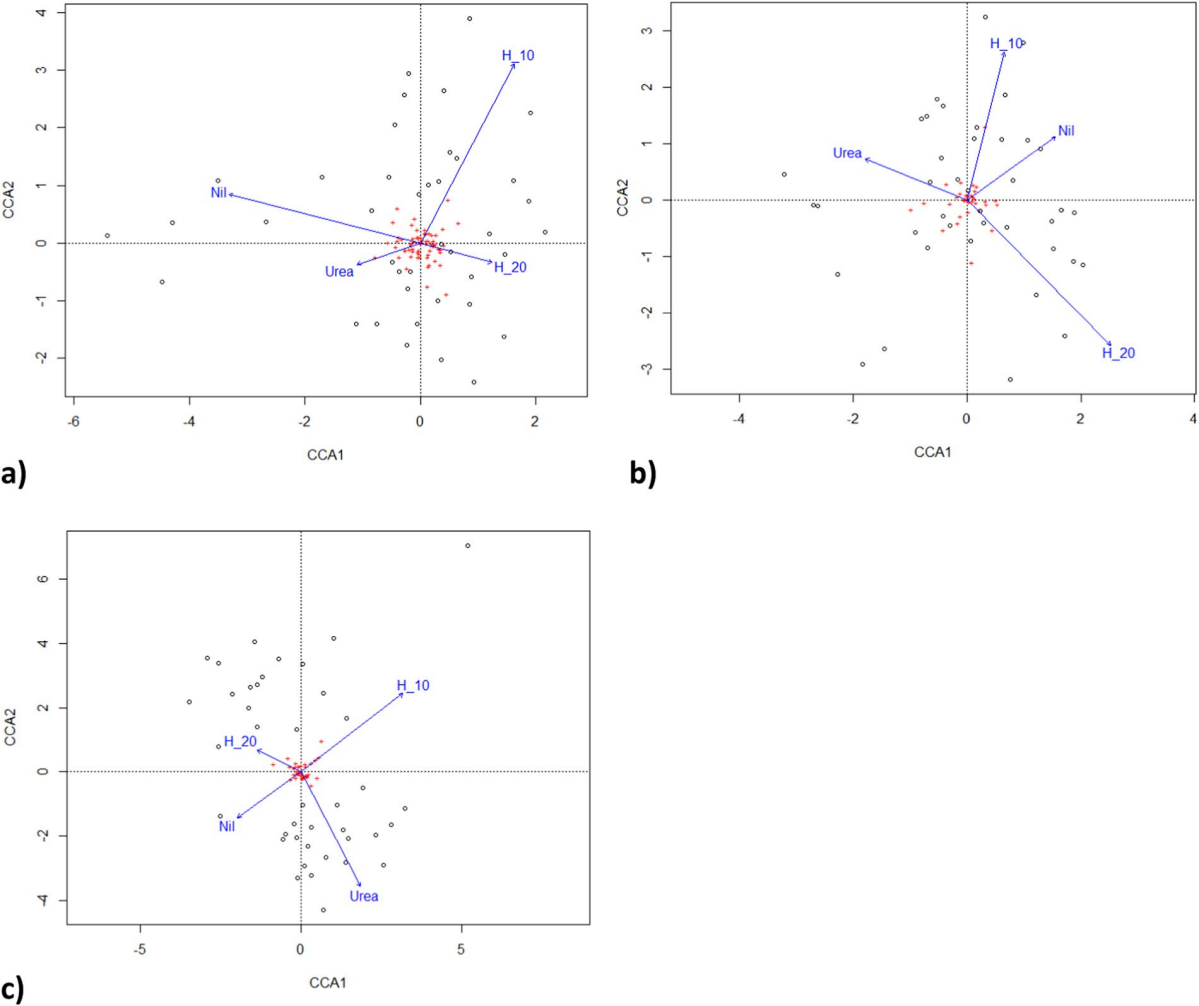
Canonical correspondence analysis of urea and humate rate (H) effect on (**a**) fungi, (**b**) bacteria, (**c**) gammaproteobacteria. Blue vector lines indicate direction and strength of fertiliser interactions, circles = fertiliser plots, crosses = taxa, H = % Humate.

Total bacterial and fungal biomass and activity varied during the first growing season (2014–15) decreasing from spring to summer then increasing in autumn (F = 5.35, P < 0.001, bacteria F = 5.25, P < 0.007, Fig. 3a,b; active bacteria F = 28.4, P < 0.0001; active fungi F = 17.84, P < 0.000;Fig. 4c,d). Rate of humate application significantly affected only fungal activity (F = 2.99, P < 0.03).

**Figure 5:**
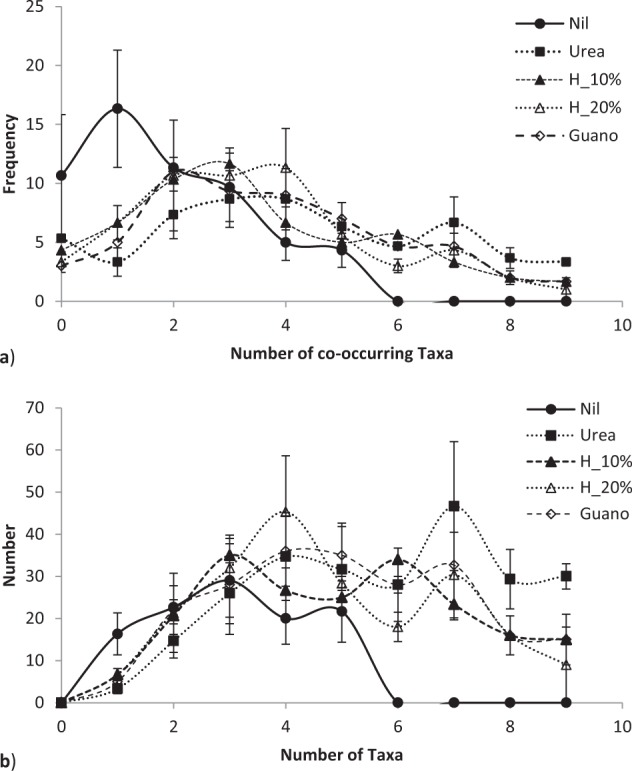
Effect of urea, urea plus humate (H) and urea plus humate and guano on (**a**) the number of co –occurring combined microbial taxa and (**b**) total number taxa per sample, mean ± standard error.

By the third growing season, in January 2017, fertiliser application differentially affected microbial populations (Fig. 4). Fertiliser significantly altered both the number of co-occurring taxa (chi-squared bacteria = 113.4, P < 0.00001; fungi = 60.4, P < 0.0067; = 76.7, P < 0.0001; Fig. 5a) and the total number of taxa (F = 2.95, P < 0.022; Fig. 5b). Fertiliser was significantly related to two specific bacteria operational taxonomic units, five fungal taxa and one gammaproteobacteria taxa at the P < 0.05 level of significance, one bacterial taxa at the P < 0.01 significance level and one fungal taxa at the P < 0.001 significance level in generalised linear models. Fertiliser also significantly affected the distribution of bacteria with ammonia oxidising genes in which occurred in all urea treatments, once in a urea plus humate plus guano treatment but were absent on all nil fertiliser, 10% and 20% urea plus humate treatments (chi-squared = 15.143, P < 0.004, Fisher’s Exact test P < 0.001). Pasture production in January was significantly related to composition of fungal taxa (multivariate general linear model, Likelihood Ratio Test LTR = 103.8, P < 0.02) and gammaproteobacteria (LRT = 60.1, P < 0.007) but not to bacteria populations (LRT = 24.8, P < 0.29).

## Discussion

N deficiency limited pasture production, as shown by the 22.9% average increase with urea across all seasons, which is consistent with previous Southland studies^54,55^ and responses on many global soils^14,16^. Humate addition increased production by a further 9.8%, a magnitude which is agriculturally significant and demonstrates superior N use efficiency of production per unit of applied N. The increase in average yield with humate was similar to the 9–14% increases reported in other New Zealand^56^ ryegrass - white clover humate trials. Ten percent humate addition gave the greatest rate response while 20% gave a slightly lower non-significant response, possibly indicating a small inhibitory rate effect or this may simply be due to experimental variability.

The effect of humate was consistent and persistent, most notably over winter in 2015 where 10% and 20% humate increased early spring production by 31% and 41% more than urea, before soil N pools were depleted and yield declined in late spring. This response, occurring when plant demand for N is increasing but soil available N is limiting, showed that humate addition resulted in enhanced supply of N and possibly other nutrients. If this was due to greater retention of summer applied N over late autumn - winter when the risk of nitrate leaching and nitrous oxide emissions are greatest due to high rainfall and soil moisture levels, low temperatures, slow plant growth and low nutrient demand^57,58^, humate may confer environmental benefit by reduce the quantity of N available for leaching or volatilisation, possibly by sequestration in microbial biomass^59,60^.

The yield increase with humate is consistent with beneficial effect of humic substance reported in other pasture species trials. Ryegrass shoot yield increased by 150% on oxisols with humic addition while red clover (*Trifolium pratense L.)* showed only a small increase, attributed to differences in P mobilisation between the species^61^. Growth of Italian ryegrass (*Lolium multiflorum* Lam.) and lucerne (*Medicago sativa* L.) was significantly increased with Australian lignite-derived humates^62^. Humic substances have significantly increased growth many other non-pasture species in many other studies ^30,35,36^. The reason for the progressive decline in relative response to fertiliser application during this trial is not known, but cannot be attributed to N accumulaion from urea as production increased in unfertilised pasture. It may be due to factors related to cessation of mechanical disturbance or enhanced biological N production.

Fertiliser significantly affected the biomass and composition of soil fungal populations in the first season though the variability associated with microscopic assessment may have masked detection of differences in other populations. Pasture yield in the third season was significantly correlated with the taxonomic diversity fungi and gammaproteobactera populations, determined by more sensitive DNA amplification, and fertiliser application was significantly related to individual fungal, bacterial and gammaproteobacterial taxa. This showed that microbial interaction is a causal factor related to plant production and that humate affected some interactions differently from urea, particularly as the effects with cannot be attributed to the quantity of macro or micronutrients humate contained. Humate acted as a bio-stimulant affecting both microbial diversity and function of both fungal and bacterial taxa. Humic substances have previously been shown to affect rhizosphere microbial populations^63–65^ and humate may act similarly to humic extracts known to increase legume nodulation and nitrogenase activity, stimulate *nod* gene expression, cell metabolism and nutrient transport^66^. Another possible mode of action is through stimulation of microbial production of plant growth regulators such as auxins, cytokinins or abscisic acid if the appropriate humic precursors are present in populations^67–69^. Humate may also affect nutrient or micronutrient availability or uptake as humic substances have been shown to increase the abundance of micronutrients, root hair development and nutrient absorptive capacity, as well as stimulating gene expression and ion uptake activity in root membranes^70–73^. Humate may also indirectly affect plant growth processes by increasing aggregate stability and improving soil sturucture^74^.

The lower yield responses with guano addition showed that the phosphorus it supplied did not increase production, indicating that N rather than P deficiency was the principal factor limiting pasture growth. The difference in response was greatest in the first growing season when guano was only applied bimonthly, and the rate of solubilisation of organic P release compared to rapid inorganic N release from urea may have affected production responses.

The general differentiation in microbial populations between urea and urea plus humate is supported by the significant difference in functional genes specific to ammonium oxidation. This shows that humate directly influences microbial metabolic N function and that plant responses cannot be solely due to carbon coating of urea granules reducing N volatilisation losses and hence improving utilisation^42,43^. The effect of humate on gammaprotepbacteria populations, ecologically important for having genes for the oxidation of nitrite and nitrate, critical processes in soil N cycling, suggests that humate may affect soil N supply and plant nutrition through this pathway.

Recommendation for the commercial applicability of humic substance has been equivocal due to inconsistency in plant field responses, uncertainty regarding modes of action, and scarcity of scientifically robust long-term field trials^35–37^. Our results support the agricultural use of humate and suggest that failure to adequately consider microbial interactions, inadequate experimental sensitivity and insufficient experimental duration may be contributory factors in negative evaluations of humic substances. Variability in individual pasture response in the first season caused us to increase main effect replication in the remaining seasons, but despite careful assessment natural field variability still remained, masking experimental sensitivity to detect subtle fertiliser effects. Our bulk sampling in the initial assessments of microbial biomass provided a general, but relatively coarse assessment of soil microbiology, rather than more focused specific assessments, e.g. from the rhizosphere of individual pasture species, which undoubtedly contributed to experimental variability, leading to the application of more precise DNA assessment. Variation in the composition and activity of humic substances has long been recognised as a major factor contributing to inconsistency in plant responses^35,75,76^ and these results with Southern Humate cannot be uncritically generalised to all humic products.

Improving the effectiveness of urea by coating with bioactive humic carbon opens new possibilities for improving global sustainable agriculture. The bio-stimulation of the soil microbiome we observed with a pasture production system would almost certainly extend to other production systems, given the beneficial responses to humic substances already recorded across a wide range of crop and horticultural species^30,35,36,37^. The low cost, resource abundance and simplicity of incorporation with humate provides a feasible, easily implementable fertiliser technology with potential to improve N efficiency for substantial agronomic and environmental benefit.

## Data Availability

The datasets generated during and/or analysed during the current study are available from the corresponding author on reasonable request.

## References

[1] World Bank, *World Development Report 2008: Agriculture for Development* (World Bank, Washington, DC (2008).

[2] Royal Society of London, Reaping the Benefits: Science and the Sustainable Intensification of Global Agriculture Royal Society, London, (2009).

[3] Tilman D, Balzer C, Hill J, Befort BL (2011). Global food demand and the sustainable intensification of agriculture. Proceedings of the National Academy of Sciences of the United States of America.

[4] McCarthy A (2018). Global food security – Issues, challenges and technological solutions. *Trends in Food Science &*. Technology.

[5] Coomes OT (2019). Leveraging total factor productivity growth for sustainable and resilient farming. Nature Sustainability.

[6] Stoate C (2001). Ecological impacts of arable intensification in Europe. Journal of Environmental Management.

[7] Foley JA (2005). Global consequences of land use. Science.

[8] Suweis S, Carr JA, Maritan A, Rinaldo A, D’Odorico P (2015). Resilience and reactivity of global food security. Proceedings of the Natural Academy of Science Proceedings of the National Academy of Sciences of the United States of America.

[9] Cottrell RS (2019). Food production shocks across land and sea. Nature Sustainability.

[10] UN General Assembly 2015. Transforming our world: the 2030 Agenda for Sustainable Development, A/RES/70/1, available at, https://www.refworld.org/docid/57b6e3e44.html [accessed 9 February 2019]

[11] Godfray HC (2010). Food security: the challenge of feeding 9 billion people. Science.

[12] Lu C, Tian H (2017). Global nitrogen and phosphorus fertilizer use for agriculture production in the past half century: shifted hot spots and nutrient imbalance. *Earth System Science*. Data.

[13] Chen Y (2019). China and India lead in greening of the world through land-use management. Nature Sustainability.

[14] Robertson GP, Vitousek PM (2009). Nitrogen in Agriculture: Balancing the Cost of an Essential Resource. Annual Review of Environment and Resources.

[15] Dawson CJ, Hilton J (2011). Fertiliser availability in a resource-limited world: Production and recycling of nitrogen and phosphorus. Food Policy.

[16] Guignard MS (2017). Impacts of nitrogen and phosphorus: From genomes to natural ecosystems and agriculture. Frontiers in Ecology and Evolution.

[17] Camargo JA, Alonso A (2006). Ecological and toxicological effects of inorganic nitrogen pollution in aquatic ecosystems: A global assessment. Environment International..

[18] International Panel on Climate Change. Climate change and land. IPCC special report, www.ipcc.ch/srccl (2019).

[19] Tilman D (2001). Forecasting agriculturally driven global environmental change. Science.

[20] Foley JA (2012). Solutions for a cultivated planet. Nature.

[21] Sylvia, D. M. Principles and Applications of Soil Microbiology. Upper Saddle River: Prentice Hall (1998).

[22] Zak DR, Holmes WE, White DC, Peacock AD, Tilman D (2003). Plant diversity, soil microbial communities, and ecosystem function: Are there any links. Ecology.

[23] Bardgett RD, Bowman WD, Kaufmann R, Schmidt SK (2005). A temporal approach to linking aboveground and belowground ecology. Trends in Ecology and. Evolution..

[24] Schimel JP, Bennett J (2004). Nitrogen mineralization: Challenges of a changing paradigm. Ecology.

[25] van der Heijden MGA, Bardgett RD, van Straalen NM (2008). The unseen majority: soil microbes as drivers of plant diversity and productivity in terrestrial ecosystems. Ecology Letters.

[26] Bengtson P, Barker J, Grayston SJ (2012). Evidence of a strong coupling between root exudation, C and N availability, and stimulated SOM decomposition caused by rhizosphere priming effects. Ecology and Evolution.

[27] Burdick EM (1965). Commercial humates for agriculture and the fertilizer industry. Economic Botany.

[28] Jansson JK, Hofmockel KS (2019). Soil microbiomes and climate change. Nature Reviews Microbiology.

[29] Ouni Y (2014). The role of humic substances in mitigating the harmful effects of soil salinity and improve plant productivity. International Journal of Plant Production.

[30] Canellas LP (2015). Humic and fulvic acids as biostimulants in horticulture. Scientia Horticulturae..

[31] Asli S, Neumann PM (2010). Rhizosphere humic acid interacts with root cell walls to reduce hydraulic conductivity and plant development. Plant and Soil.

[32] de Santiago A, Exposito A, Quintero JM, Carmona E, Delgado A (2010). Adverse effects of humic substances from different origin on lupin as related to iron sources. Journal of Plant Nutrition.

[33] Hartz TK, Bottoms G (2010). Humic substances generally ineffective in improving vegetable crop nutrient uptake or productivity. Hortscience.

[34] Mahoney KJ, McCreary C, Depuydt D, Gillard CL (2017). Fulvic and humic acid fertilizers are ineffective in dry bean. Canadian Journal of Plant Science.

[35] Olk DC, Dinnes DL, Scoresby JR, Callaway CR, Darlington JW (2018). Humic products in agriculture: potential benefits and research challenges—a review. Journal of Soils and Sediments.

[36] Quilty JR, Cattle SR (2011). Use and understanding of organic amendments in Australian agriculture: a review. Soil Research.

[37] Billingham KL (2012). Humic products – potential or presumption for agriculture? Can humic products improve my soil?. Proceedings of the 27th Annual Conference of the Grasslands Society of New South Wales.

[38] Bell NG, Michalchuk AA, Blackburn JW, Graham MC, Uhrin D (2015). Isotope-Filtered 4D NMR Spectroscopy for Structure Determination of Humic Substances. Angewandte Chemie International Edition English.

[39] Gerke. G (2018). Concepts and misconceptions of humic substances as the stable part of organic matter: a review. Agronomy.

[40] Shah ZH (2018). Humic Substances: Determining Potential Molecular Regulatory Processes in. Plants. Frontiers in Plant Science.

[41] Kleber M (2011). Old and stable soil organic matter is not necessarily chemically recalcitrant: implications for modelling concepts and temperature sensitivity. Global Change Biology.

[42] Azeem, B., KuShaari, K. Z., Man, Z. B., Basit, A., & Trinh, T.H. Review on materials & methods to produce controlled release coated urea fertilizer. *Journal of Controlled Release* **181**, 11–21 10.1016/j.jconrel.2014.02.020 24593892

[43] Trinh TH, Kushaari K, Shuib AS, Ismail L, Azeem B (2015). Modelling the release of nitrogen from controlled release fertiliser: Constant and decay release. Biosystems Engineering.

[44] Li Y (2018). Synthesis and performance of bio-based epoxy coated urea as controlled release fertilizer. Progress in Organic Coatings.

[45] Hewitt, A. E. 1992. New Zealand Soil Classification. DSIR Land Resources Scientific Report 19.

[46] Klein DA, Paschke MW (2000). A soil microbial community structural-functional index: the microscopy-based total/active/active fungal/bacterial (TA/AFB) biovolumes ratio. Applied Soil Ecology.

[47] Muyzer G, de Waal E, Uitterlinden AG (1993). Profiling complex microbial populations by denaturing gradient gel electrophoresis analysis of polymerase chain reaction-amplified genes coding for 16S rRNA. Applied and Environmental Microbiology.

[48] Mühling M, Woolven-Allen J, Murrell J (2008). C, & Joint, I. Improved group-specific PCR primers for denaturing gradient gel electrophoresis analysis of the genetic diversity of complex microbial communities. ISME Journal..

[49] Vainio EJ, Hantula J (2000). Direct analysis of wood-inhabiting fungi using denaturing gradient gel electrophoresis of amplified ribosomal DNA. Mycological Research.

[50] Shiomura Y, Morimoto S, Hoshino YT, Uchida Y, Akiyama H, Hayatsu M (2012). Comparison among *amoA* primers suited for quantification and diversity analyses of ammonia oxidising bacteria in Soil. Microbes and Environments.

[51] R Core Development Team 2017. *R: A Language and Environment for Statistical Computing*. R Foundation for Statistical Computing. URL, https://www.R-project.org/

[52] Oksanen, J. *et al*. vegan: Community Ecology Package. R package version 2.5-6, https://CRAN.R-project.org/package=vegan (2019).

[53] Wang Y, Nauman U, Wright ST, Warton D (2012). I. mvabund- a R package for model –based analysis of multivariate abundance data. Methods in Ecology and Evolution.

[54] Risk, W. H. Use of nitrogen fertilisers on the Southland plains. In: Lynch P. B., editor. Nitrogen fertilisers in New Zealand agriculture. Wellington: New Zealand Institute of Agricultural Science; p 149 – 158 (1982).

[55] Smith LC, Morton JD, Catto WD, Trainor KD (2000). Nitrogen responses on pasture in the southern South Island of New Zealand. Proceedings of the New Zealand Grasslands Association.

[56] Schofield. P., Watt, N., & Schofield, M. Using Humic compounds to improve efficiency of fertiliser nitrogen. In: Currie LD, Christensen CL. editors. Advanced Nutrient Management: Gains from the Past - Goals for the Future. *Massey University, Palmerston North: Fertilizer and Lime Research Centre Occasional Report No*. 25 (2013).

[57] White RE, Wellings SR, Bell JP (1983). Seasonal variations in nitrate leaching in structured clay soils under mixed land use. Agricultural Water Management.

[58] Butterbach-Bahl K, Baggs EM, Dannenmann M, Kiese R, Zechmeister-Boltenstern S (2013). Nitrous oxide emissions from soils: how well do we understand the processes and their controls?. Philosophical Transactions Royal Society London B Biological Sciences.

[59] Simpson AC, Zabowski D, Rochefort RM, Edmonds RL (2019). Increased microbial uptake and plant nitrogen availability in response to simulated nitrogen deposition in alpine meadows. Geoderma.

[60] Lipson DA, Schmidt SK, Russell K, Monson RK (1999). Links between microbial population dynamics and nitrogen availability in an alpine ecosystem. Ecology.

[61] Gerke J, Meyer U, Römer W (1995). Phosphate, Fe and Mn uptake of N_2_ fixing red clover and ryegrass from an Oxisol as affected by P and model humic substances application. 1. Plant parameters and soil solution composition. Journal of Plant Nutrition and Plant Science.

[62] Little KR, Rose MT, Jackson WR, Cavagnaro TR, Patti. AF (2014). Do lignite-derived organic amendments improve early-stage pasture growth and key soil biological and physicochemical properties? *Crop and Pasture*. Science.

[63] Pinton, R., Cesco. S., & Varanini, Z. Role of humic substances on the rhizosphere. In: Senesi N, Xing B, Huang PM. editors. Biophysico-Chemical Processes Involving Natural Nonliving Organic Matter in Environmental Systems. Hoboken (NJ): John Wiley & Sons. p. 341–359 (2009).

[64] Van Trump JI, Sun Y, Coates JD (2016). Microbial interactions with humic substances. Advances in Applied Microbiology.

[65] Lipcznska-Kochany E (2018). Humic substances, their microbial interactions and effects on biological transformations of organic pollutants in water and soil: A review. Chemosphere.

[66] Gao TG (2015). Nodulation Characterization and Proteomic Profiling of Bradyrhizobium liaoningense CCBAU05525 in Response to Water-Soluble Humic Materials. Scientific Reports.

[67] Nardi S, Pizzeghello D, Muscolo A, Vianello A (2002). Physiological effects of humic substances on higher plants. Soil Biology and Biochemistry.

[68] Chen Y, Clapp CE, Magen H (2004). Mechanisms of plant growth stimulation by humic substances: The role of organo-iron complexes. Soil Science and Plant Nutrition.

[69] Muscolo A, Sidari M, Nardi S (2013). Humic substance: relationship between structure and activity. Deeper information suggests univocal findings. Journal of Geochemical Exploration.

[70] Kalis EJ, Temminghoff EJ, Weng L, van Riemsdijk WH (2006). Effects of humic acid and competing cations on metal uptake by *Lolium perenne*. Environmental Toxicology and Chemistry.

[71] Mora V (2010). Action of humic acid on promotion of cucumber shoot growth involves nitrate-related changes associated with the root-to-shoot distribution of cyto-kinins, polyamines and mineral nutrients. Journal of Plant Physiology.

[72] Maibodi ND, Kafi M, Nikbakht A, Rejali F (2015). Effect of Foliar Applications of Humic Acid on Growth, Visual Quality, Nutrients Content and Root Parameters of Perennial Ryegrass (*Lolium Perenne* L.). Journal of Plant Nutrition.

[73] Nardi S, Pizzeghello D, Schiavon M, Ertani A (2016). Plant biostimulants. Physiological responses induced by protein hydrolyzed-based products and humic substances in plant metabolism. Scientica Agricola.

[74] Imbufe A (2005). Effects of potassium humate on aggregate stability of two soils from Victoria Australia. Geoderma.

[75] Lobartini JC (1992). The geochemical nature and agricultural importance of commercial humic matter. Science of the Total Environment.

[76] Rose MT, Patti AF, Little KR, Brown AL (2014). A meta-analysis and review of plant-growth response to humic substances: practical implications for agriculture. Advances In Agronomy.

